# OBSERVING SUPPORT INTERACTIONS BETWEEN PARENTS AND CHILDREN IN ADVANCED AGE

**DOI:** 10.1093/geroni/igz038.2480

**Published:** 2019-11-08

**Authors:** Claudia Meystre, Daniela Jopp, Joëlle Darwiche, Kathrin Boerner, Dario Spini

**Affiliations:** 1 University of Lausanne, Institute of Psychology, Lausanne, Vaud, Switzerland; 2 University of Lausanne, Lausanne, Switzerland, Switzerland; 3 University of Massachusetts Boston, Boston, Massachusetts, United States; 4 University of Lausanne, Swiss National Centre of Competence in Research LIVES, Lausanne, Vaud, Switzerland

## Abstract

Studies on support exchanges in older parent-child dyads have so far not used observational approaches. Rather, they have mostly relied on self-report/questionnaire approaches. However, support exchanges represent a dyadic phenomenon that goes beyond individual perspectives on the quality of support; thus, self-reports offer only a part of the picture and are subject to bias (e.g., memory bias). In contrast, observations are better suited to capture specific support behaviors and allow for studying the dyad “in action.” Our study purpose was to examine mutual support during interactions between older adults and their children, and to investigate the links of support behaviors to relationship quality and health. Fifty dyads living in Switzerland, composed of individuals aged 70 and over and the child involved in their care, participated in a standardized interaction task: The parent described and discussed a personally challenging situation with the child for ten minutes; then roles were reversed. The videotaped interaction was analyzed using the Social Support Behavior Code Adapted for Elders (Meystre et al., submitted). Relationship quality and subjective health were assessed via standardized questionnaires. Various types of support were observed, ranging from positive to negative. Children provided more informational support than parents. Dyads with poor relationship quality showed more informational support. Better participant health was associated with fewer negative behaviors. In sum, observing the dyads in real time offered unique insights into interacting patterns of support among older parents and their children, and enabled us to capture the nature of their relationship above and beyond individual self-reports.

